# Matrix metalloproteinase-12 by M2 macrophages induced epithelial to mesenchymal transition in chronic rhinosinusitis with nasal polyps

**DOI:** 10.1371/journal.pone.0313097

**Published:** 2024-12-31

**Authors:** Joo-Hoo Park, Jae-Min Shin, Hyun-Woo Yang, Tae Hoon Kim, Seung Hoon Lee, Ok Sarah Shin, Il-Ho Park

**Affiliations:** 1 Upper Airway Chronic Inflammatory Diseases Laboratory, Korea University College of Medicine, Seoul, Republic of Korea; 2 Medical Device Usability Test Center, Korea University Guro Hospital, Seoul, Republic of Korea; 3 Department of Medicine, Korea University College of Medicine, Seoul, Republic of Korea; 4 Department of Otorhinolaryngology-Head and Neck Surgery, Korea University College of Medicine, Seoul, Republic of Korea; 5 BK21 Graduate Program, Department of Biomedical Sciences, College of Medicine, Korea University Guro Hospital, Seoul, Republic of Korea; Huashan Hospital Fudan University, CHINA

## Abstract

Th2 inflammation and epithelial-mesenchymal transition (EMT) play crucial roles in the pathophysiology of chronic rhinosinusitis with nasal polyps (CRSwNP). This study aimed to investigate the hypothesis that MMP-12, produced by M2 macrophages, induces EMT in nasal epithelial cells, thereby contributing to airway inflammation and remodeling in CRSwNP. The expression levels of MMP-12 were measured by RT-PCR in CRS nasal mucosa and THP-1 cells. mRNA and protein levels of E-cadherin, vimentin, α-SMA, and fibronectin were determined using RT-PCR, western blotting, and immunofluorescence staining in primary nasal epithelial cells and air-liquid interface culture. The expression of MMP-12 was significantly increased in CRSwNP and M2-like THP-1 cells. In co-culture with primary nasal epithelial cells and M2-like THP-1 cells, E-cadherin expression was inhibited, and fibronectin, vimentin, and α-SMA expression were increased. MMP-12 decreased E-cadherin but induced fibronectin, vimentin, and α-SMA mRNA and protein expression in primary nasal epithelial cells and air-liquid interface culture. MMP408, an MMP-12 inhibitor, inhibited EMT-related factors. These findings suggest that MMP-12 expression in M2 macrophages induces EMT in nasal epithelial cells and may contribute to the pathogenesis of CRSwNP.

## Introduction

Chronic rhinosinusitis (CRS), one of the most common chronic inflammatory diseases, is characterized by symptoms such as rhinorrhea, postnasal drip, nasal obstruction or congestion, sinus pain or pressure, and anosmia or hyposmia lasting for at least 12 weeks [1]. CRS is a heterogeneous disease with diverse clinical presentations and complex underlying pathophysiology. Current classification systems for CRS are primarily based on clinical criteria, such as the presence or absence of nasal polyps, and recent efforts have been made to define endotypes of CRS based on fundamental immune and inflammatory mechanisms, such as Th1/Th2/Th17 immune responses and eosinophilic inflammation [2]. However, some limitations have been observed in the clinical usefulness of these classification systems. Therefore, additional auxiliary endotype classification systems that can further differentiate CRS into subgroups may increase the clinical usefulness by allowing for more targeted treatment approaches.

The pathogenesis of CRS is complex and multifactorial, involving a range of inflammatory and immune mechanisms. Macrophages are one of the key cell types involved in the immune response in CRS, and have been shown to play a critical role in the regulation of inflammation and tissue repair [3, 4]. While macrophages are essential for host defense and tissue homeostasis, their dysregulation has been implicated in the pathogenesis of CRS [5, 6]. In particular, aberrant polarization of macrophages towards the M2 phenotype has been associated with disease progression and tissue remodeling in CRS [7]. Understanding the role of macrophages in the pathogenesis of CRS could provide new insights into disease mechanisms and help to identify novel therapeutic targets.

Epithelial-mesenchymal transition (EMT) is a biological process that allows epithelial cells to lose their cell-cell adhesion and polarity, and to gain mesenchymal properties [8]. EMT is involved in the pathogenesis of CRS, a common inflammatory disorder of the nasal and paranasal sinus mucosa [9]. Recent studies have suggested that EMT can be induced by the presence of M2 macrophages, which are a subset of macrophages with anti-inflammatory and tissue remodeling properties [10]. M2 macrophages are known to secrete various growth factors, cytokines, and extracellular matrix proteins that can promote EMT in epithelial cells [11]. Understanding the relationship between M2 macrophages and EMT is important for developing new therapeutic approaches for inflammatory airway diseases which are characterized by abnormal EMT. Recently, it has been suggested that tissue remodeling including EMT is related to disease resistance in CRS. However, the roles and underlying mechanisms of tissue remodeling, including EMT, in epithelium of CRS patients are unclear.

Matrix metalloproteinase-12 (MMP-12), a key enzyme produced by M2 macrophages, has been identified as a significant mediator in this context due to its role in extracellular matrix degradation and tissue remodeling. MMP-12 is known to promote EMT by cleaving E-cadherin, a major epithelial marker, and increasing the expression of mesenchymal markers such as vimentin and fibronectin [12]. In CRS, elevated levels of MMP-12 have been associated with disease severity, suggesting its potential role in the progression of the disease through EMT induction [13].

Based on this evidence, we hypothesize that MMP-12 produced by M2 macrophages contributes to the pathogenesis of CRSwNP by inducing EMT in nasal epithelial cells. This induction of EMT may lead to increased tissue remodeling and fibrosis, which are hallmarks of CRSwNP. The aim of this study is to investigate the expression level of MMP-12 in M2 macrophages in CRS patients, evaluate the potential of MMP-12 to induce EMT in nasal epithelial cells, and elucidate the molecular mechanisms underlying the induction of EMT by MMP-12 in nasal epithelial cells.

## Methods

### Patients and specimens

Normal uncinate processes were collected from patients who underwent surgery for pituitary tumors, and uncinate processes and nasal polyps of disease groups were collected from patients diagnosed with chronic rhinosinusitis (CRS) who underwent endoscopic sinus surgery. The specimens were collected between May 11, 2023, and December 14, 2023. To exclude any confounding factors unrelated to the research, patients who had used systemic or topical steroids, macrolides, or antibiotics for at least 4 weeks before surgery, as well as patients with other upper respiratory tract diseases, were excluded. The diagnostic criteria for patients with chronic rhinosinusitis (CRS) were applied in accordance with the 2020 European Position Paper on Rhinosinusitis and Nasal Polyps. Detailed clinical characteristics of the patients are available in Table 1. This study was carried out with appropriate ethical approval granted by the Korea University Medical Center Institutional Review Board (approval number: 2023GR0179). In adherence to the principles of the Declaration of Helsinki, written informed consent was obtained from all participating patients. As this study involved a retrospective analysis of medical records or archived samples, all data were fully anonymized before access.

**Table 1:** Clinical characteristics of subjects (N = 37).

Characteristics	Healthy UP (n = 4)	CRSsNP-UP (n = 10)	CRSwNP-UP (n = 10)	CRSwNP-NP (n = 13)
**Number of women/men**	3/1	3/7	2/8	4/9
**Age (years, mean ± SD)**	40.3 ± 5.7	46.6 ± 4.2	44.6 ± 6.7	49.3 ± 7.1
**Asthma**	0	0	0	0
**Allergic rhinitis**	0	0	0	0
**Lund-Mackay**	0.75	12.9	16.2	15
**CT score**				

UP, uncinate process; NP, nasal polyps; CRSsNP, chronic rhinosinusitis without nasal polyps; CRSwNP, chronic rhinosinusitis with nasal polyps; SD, standard deviation.

### Cell cultures

Human airway epithelial cell line A549 (ATCC CCL-185) was purchase from the Korean Cell Line Bank (KCLB, Seoul, Korea), and cultured in RPMI 1640 medium (hyclone, Logan, UT, USA) containing 10% fetal bovine serum (FBS, Gibco, Grand Island, NY, USA), 1% penicillin/streptomycin (Invitrogen, Carlsbad, CA, USA) in a humidified incubator at 37°C and 5% CO_2_. Approval from the Korea University Medical Center Institutional Review Board (approval number: 2023GR0179) and written informed consent in accordance with the Declaration of Helsinki were obtained to collect primary nasal epithelial cells by scraping the mid-inferior turbinate with a brush and immediately placing them in RPMI 1640 medium containing 1% penicillin/streptomycin for this study. After centrifugation at 1500 rpm for 3 minutes, the red blood cells were removed using RBC lysis buffer (Sigma-Aldrich, St. Louis, MO, USA). Human primary nasal epithelial cells were cultured in PneumaCult-Ex Plus Medium (Stemcell Technologies, Vancouver, Canada) for 3 days in collagen type 1 coated dishes (Corning Incorporated, Corning, NY, USA) [14].

Primary nasal epithelial cells were cultured at the air-liquid interface (ALI) to mimic the in vivo conditions of the nasal epithelium. Cells were seeded on collagen-coated transwell inserts (Corning, NY, USA) at a density of 1 × 10^5^ cells per well and maintained in PneumaCult-Ex Plus Medium (Stemcell Technologies) in a submerged state for 7 days to allow full confluence. After reaching confluence, the medium was removed from the apical chamber, and the cells were exposed to air while the basal chamber continued to be fed with PneumaCult-ALI Maintenance Medium (Stemcell Technologies) supplemented with hydrocortisone, heparin, and other growth factors. The ALI culture was maintained for an additional 21 days, with medium changes every 2–3 days.

Co-culture of macrophages and epithelial cells was performed using a transwell system. Briefly, epithelial cells were seeded in the upper chamber and allowed to attach overnight. The following day, THP-1 cells were differentiated into macrophages using phorbol 12-myristate 13-acetate (PMA, 100nM) and added to the lower chamber containing a polycarbonate membrane with 0.4 μm pores. To differentiate THP-1 cells into M1 macrophages, cells are treated with 100 ng/mL of lipopolysaccharide (LPS) and 20 ng/mL of interferon-gamma (IFN-γ) for 24 hours. On the other hand, to differentiate THP-1 cells into M2 macrophages, cells are treated with 20 ng/mL of interleukin-4 (IL-4) and interleukin-13 (IL-13) for 24 hours. The co-culture was maintained for 24 hours to allow for interaction between the two cell types while preventing direct contact. After 24 hours, the upper chamber was removed, and the epithelial cells were collected for further analysis. Control groups included epithelial cells cultured alone and macrophages cultured alone. The co-culture experiment was performed in triplicate.

### Real-time PCR

Total RNA was isolated from the samples using TRIzol reagent (Invitrogen). Then, 2 μg of RNA was reverse transcribed into cDNA using M-MLV reverse transcriptase (Invitrogen) and oligo (dT) as the primers. The synthesized cDNA and primers specific to the target RNA were mixed with Power SYBR Green PCR Master Mix (Applied Biosystems). Real-time PCR was performed using the Quantstudio3 to measure the expression level of the target gene. The cycling conditions included an initial denaturation step, followed by 40 cycles of amplification and melting curve analysis. The forward and reverse primers used for real-time PCR are listed in Table 2. The results were analyzed using the ΔΔCt method and normalized to the expression of a reference gene.

**Table 2:** Sequences of PCR primers.

Gene Name		Sequences (quantitative RT-PCR)
***CD206***	Forward	5ʹ-GGG TTG CTA TCA CTC TCT ATG C-3ʹ
	Reverse	5ʹ-TTT CTT GTC TGT TGC CGT AGT T-3ʹ
***CD163***	Forward	5ʹ-TTT GTC AAC TTG AGT CCC TTC AC-3ʹ
	Reverse	5ʹ-TCC CGC TAC ACT TGT TTT CAC-3ʹ
***MMP-12***	Forward	5ʹ-GAT CCA AAG GCC GTA ATG TTC C-3ʹ
	Reverse	5ʹ-TGA ATG CCA CGT ATG TCA TCA G-3ʹ
***TNFα***	Forward	5ʹ-GAG GCC AAG CCC TGG TAT G-3ʹ
	Reverse	5ʹ-CGG GCC GAT TGA TCT CAG C-3ʹ
***IL-6***	Forward	5ʹ-ACT CAC CTC TTC AGA ACG AAT TG-3ʹ
	Reverse	5ʹ-CCA TCT TTG GAA GGT TCA GGT TG-3-3ʹ
***IL-1β***	Forward	5ʹ-TTC GAC ACA TGG GAT AAC GAG G-3ʹ
	Reverse	5ʹ-TTT TTG CTG TGA GTC CCG GAG-3ʹ
***CXCL10***	Forward	5ʹ-GTG GCA TTC AAG GAG TAC CTC-3ʹ
	Reverse	5ʹ-TGA TGG CCT TCG ATT CTG GAT T-3ʹ
***CCL22***	Forward	5ʹ-ATT ACG TCC GTT ACC GTC TGC-3ʹ
	Reverse	5ʹ-TCC CTG AAG GTT AGC AAC ACC-3ʹ
***Dectin-1***	Forward	5ʹ-GGA AGC AAC ACA TTG GAG AAT GG-3ʹ
	Reverse	5ʹ-AGA ACC CCT GTG GTT TTG ACA-3ʹ
***E-cadherin***	Forward	5′-TGC TCT TGC TGT TTC TTC GG-3′
	Reverse	5′-TGC CCC ATT CGT TCA AGT AG-3′
***Vimentin***	Forward	5′-CTC TTG CTC TGG GCT TCA TC-3′
	Reverse	5′-CTC TTG CTC TGG GCT TCA TC-3′
***α-SMA***	Forward	5′-GGC TCT GGG CTC TGG GCT TCA TC-3′
	Reverse	5′-CTC TTG CTC TGG GCT TCA TC-3′
***Fibronectin***	Forward	5′-CTT TGG TGC AGC ACA ACT TC-3′
	Reverse	5′-CCT CCT CGA GTC TGA ACC AA-3′
***GAPDH***	Forward	5′-GTG GAT ATT GTT GCC ATC AAT GAC C-3′
	Reverse	5′-GCC CCA GCC TTC TTC ATG GTG GT-3′

### Western blot analysis

The western blot analysis method was previously described [14]. A549 and primary nasal epithelial cells were lysed using RIPA buffer (Cell Signaling Technology, Danvers, MA, USA) for isolation of protein extracts. The extracted protein concentrations were quantitated using the Bradford assay reagent (Bio-Rad). The equal amounts of protein from each sample with 5X SDS-PAGE loading buffer (Biosesang, Gyeonggi-do, South Korea) were separated by 10% SDS-PAGE gel and transferred onto polyvinyl difluoride membranes (Millipore Inc., Billerica, MA, USA). The membranes were placed in 5% skim milk to block non-specific binding for 1 h at room tempferature. After washing with TBST (Tris-buffered saline, 0.1% Tween 20), the membranes were incubated with primary antibodies overnight at 4°C. The primary antibodies included anti-MMP-12 (1:1000, R&D Systems, Minneapolis, MN, USA), anti-α-SMA, (1: 10000, Abcam, Cambridge, MA, USA), anti-E-cadherin, anti-fibronectin, anti-GAPDH (1: 1000, Santa Cruz Biotechnology, Inc., Santa Cruz, CA, USA), anti-vimentin (1:1000, Cell Signaling Technology, Danvers, MA, USA). Following incubation of primary antibody, HRP-conjugated anti-mouse or anti-rabbit antibodies (1:10000, Vector Laboratories, Burlingame, CA, USA) were added to the membrane for 1 h. Immunoblot signals representing the specific proteins were detected using the enhanced chemiluminescence (ECL) detection system (Pierce, Rockford, IL). The PageRuler™ Plus Prestained Protein Ladder (10 to 250 kDa; Thermo Scientific™) was used as a molecular weight marker. The images were analyzed using ImageJ software (NIH, Rockville, MD, USA) for quantification of band intensity.

### Short interfering RNA transfection

Transfection of MMP-12 siRNA (Bioneer, Daejeon, Republic of Korea) was performed using Lipofectamine^TM^ RNAiMax (Invitrogen) according to the manufacturer’s instructions. Briefly, THP-1 cells were seeded in 6-well plates and grown to 50–60% confluence. MMP-12 siRNA and Lipofectamine^TM^ RNAiMax reagent were separately diluted in Opti-MEM reduced serum medium (Gibco) and mixed gently before incubation at room temperature for 5 minutes. The diluted MMP-12 siRNA was then added to the diluted Lipofectamine^TM^ RNAiMax reagent and the mixture was incubated for an additional 20 minutes at room temperature to allow complex formation. The resulting complex was added to the cells and incubated for 6 hours at 37°C in a CO2 incubator. After 6 hours, the transfection medium was replaced with fresh complete medium and the cells were cultured for an additional 48–72 hours before further experiments were performed. The efficiency of MMP-12 knockdown was confirmed by real-time PCR and Western blot analysis.

### Immunofluorescence staining

For immunofluorescence staining, cells grown on coverslips were fixed in 4% paraformaldehyde for 30 minutes at room temperature to preserve cellular structures and then permeabilized with 0.1% Triton X-100 in phosphate-buffered saline (PBS) for 10 minutes to allow antibody access to intracellular proteins. After permeabilization, cells were blocked with 5% bovine serum albumin (BSA) in PBS for 1 hour to prevent non-specific binding of antibodies. The cells were then incubated with primary antibodies against CD206, MMP-12, E-cadherin, or Vimentin (1:100 dilution in blocking buffer) at 4°C overnight. Following primary antibody incubation, cells were washed with PBS and incubated with fluorescently-labeled secondary antibodies, goat anti-mouse IgG (H+L) Alexa 488 or goat anti-rabbit IgG (H+L) Alexa 555 (Invitrogen, Carlsbad, CA, USA; 1:200 dilution), for 1 hour at room temperature. Nuclei were counterstained with DAPI (Invitrogen, Carlsbad, CA, USA) for 10 minutes. After extensive washing with PBS to remove unbound antibodies, coverslips were mounted on glass slides using an anti-fade mounting medium (Vector Laboratories, Burlingame, CA, USA). Images of the stained cells were captured using a confocal laser scanning microscope LSM700 (Zeiss, Oberkochen, Germany).

### Statistical analysis

The data are presented as mean ± standard deviation and represent results obtained from a minimum of three independent experiments. Before applying parametric tests, the normality of data distribution was assessed using the Shapiro-Wilk test, and homogeneity of variances was verified using Levene’s test. Statistical analysis was then carried out using GraphPad Prism 5 (Graph Pad Software, San Diego, CA, USA). Depending on the results of these preliminary tests, appropriate parametric tests, such as unpaired t-tests or one-way analysis of variance (ANOVA), followed by Tukey’s post-hoc test, was employed. Each experimental condition was tested in triplicate, and statistical significance was defined as P-values less than 0.05. Additionally, Pearson’s correlation test was applied to assess the relationships between variables where applicable, with details provided on the data characteristics and the rationale for choosing this test.

## Results

### MMP-12 expression by M2-macrophage was increased in CRS patients and correlated with severity

**Fig 1:**
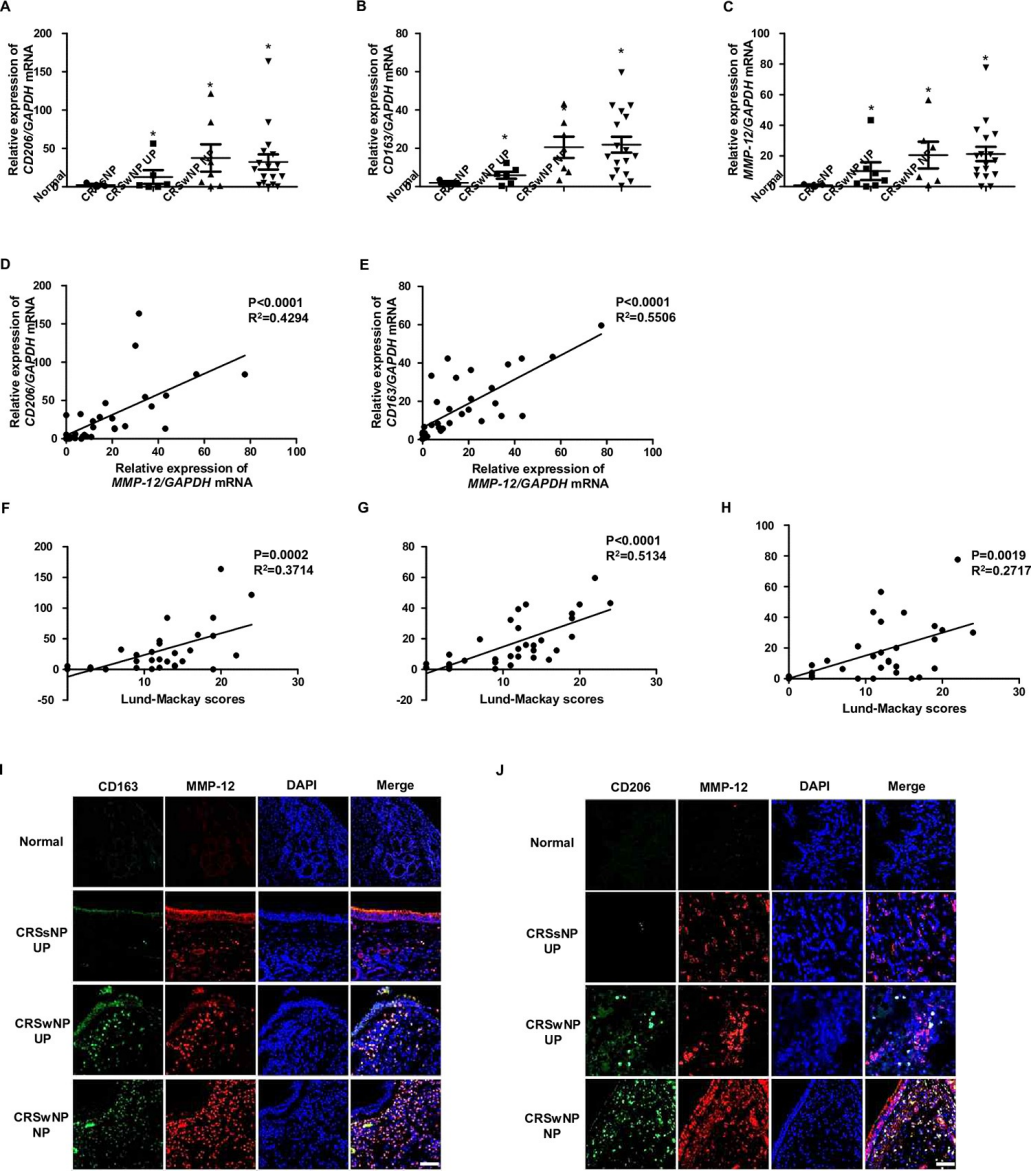
Expression of CD206, CD163 and MMP-12 in different types of nasal tissues from patients with CRS. mRNA expression of (A) CD206, (B) CD163 and (C) MMP-12 in normal UP, CRSsNP UP, CRSwNP UP, and CRSwNP NP tissues. (D) Correlation analysis between the mRNA expression of CD206 and MMP-12, (E) as well as between the mRNA expression of CD163 and MMP-12. Correlation analysis between the mRNA expression of (F) CD206, (G) CD163 and (H) MMP-12, and the Lund-Mackay score. Immunofluorescence analysis showing (I) CD206-positive cells (green), (J) CD163-positive cells (green) and MMP-12 expression (red). The results are presented as mean ± SEM. *P < 0.05 compared to normal UP. Data are presented as the mean ± SD of three independent experiments.

The mRNA expression of CD206 and CD163, known as markers of M2 macrophages, as well as the expression of mRNA MMP12, were examined in normal uncinate process (UP), CRS without nasal polyp (CRSsNP) UP, CRS with nasal polyp (CRSwNP) UP, and CRSwNP NP tissues. The results showed that both CD206, CD163 and MMP-12 were significantly increased in CRSsNP UP, CRSwNP UP, and CRSwNP NP tissues, with the greatest increase observed in CRSwNP UP tissues (Fig 1A–1C). The correlation between the expression of CD206 and MMP-12, as well as between the expression of CD163 and MMP-12, was investigated. Both the expression of CD206 and MMP-12, and the expression of CD163 and MMP-12 showed significantly positive correlations. (Fig 1D and 1E). Furthermore, CD206, CD163 and MMP-12 expression showed a significant correlation with the severity of disease as measured by the Lund-Mackay score (Fig 1F–1H). To determine whether MMP-12 is expressed in CD206-positive cells, immunofluorescence was performed. The results showed that CD206-positive cells were most abundant in the CRSwNP NP tissue, and MMP-12 was also most strongly expressed in these cells. Furthermore, MMP-12 was found to be expressed in CD206-positive cells (Fig 1F). Similarly, CD163-positive cells showed a pattern similar to CD206-positive cells, with the highest abundance observed in the CRSwNP NP tissue, and MMP-12 co-localized with CD163-positive cells (Fig 1I and 1J). These findings collectively indicate that both CD206 and CD163 are positively correlated with MMP-12 expression, suggesting the potential role of M2 macrophages and their associated markers in the regulation of MMP-12 in chronic rhinosinusitis.

### Characterization of polarized THP-1 macrophages

**Fig 2:**
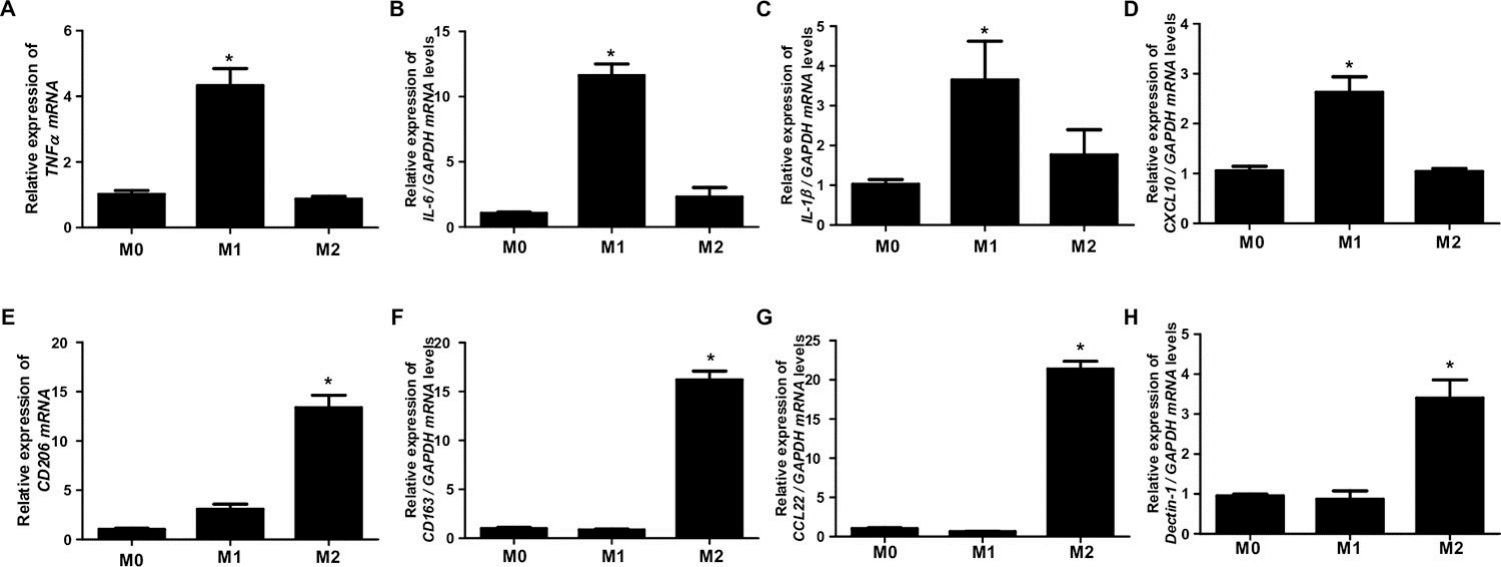
Characterization of polarized THP-1 macrophages. (A-D) THP-1 cells were differentiated into M1 macrophages using PMA and then stimulated with LPS and IFN-γ for 24 hours. The expression of TNFα, IL-6, IL-1β, and CXCL10 were then assessed by real-time PCR. (E-H) THP-1 cells were differentiated into M2 macrophages using IL-4 and then stimulated with IL-13 for 24 hours. The expression of CD206, CD163, CCL22, and Dectin1 were then assessed by real-time PCR. *p < 0.05 compared to undifferentiated THP-1 cells. Data are presented as the mean ± SD of three independent experiments.

To characterize polarized THP-1 macrophages, THP-1 cells were differentiated into M1 and M2 macrophages, and the expression of markers specific to each phenotype was assessed. TNFα, IL-6, IL-1β, and CXCL10, which are markers of M1 macrophages, were increased in THP-1 cells differentiated into M1 macrophages, but not in those differentiated into M2 macrophages (Fig 2A–2D). In contrast, CD206, CD163, CCL22, and Dectin1, which are markers of M2 macrophages, were increased in THP-1 cells differentiated into M2 macrophages, but not in those differentiated into M1 macrophages (Fig 2E–2H). These findings confirm successful differentiation of THP-1 cells into polarized M1 and M2 macrophages, and suggest that these cells can be used to study the function of macrophage subtypes in various diseases.

### M2 macrophage induced EMT process in A549 and PNECs

**Fig 3:**
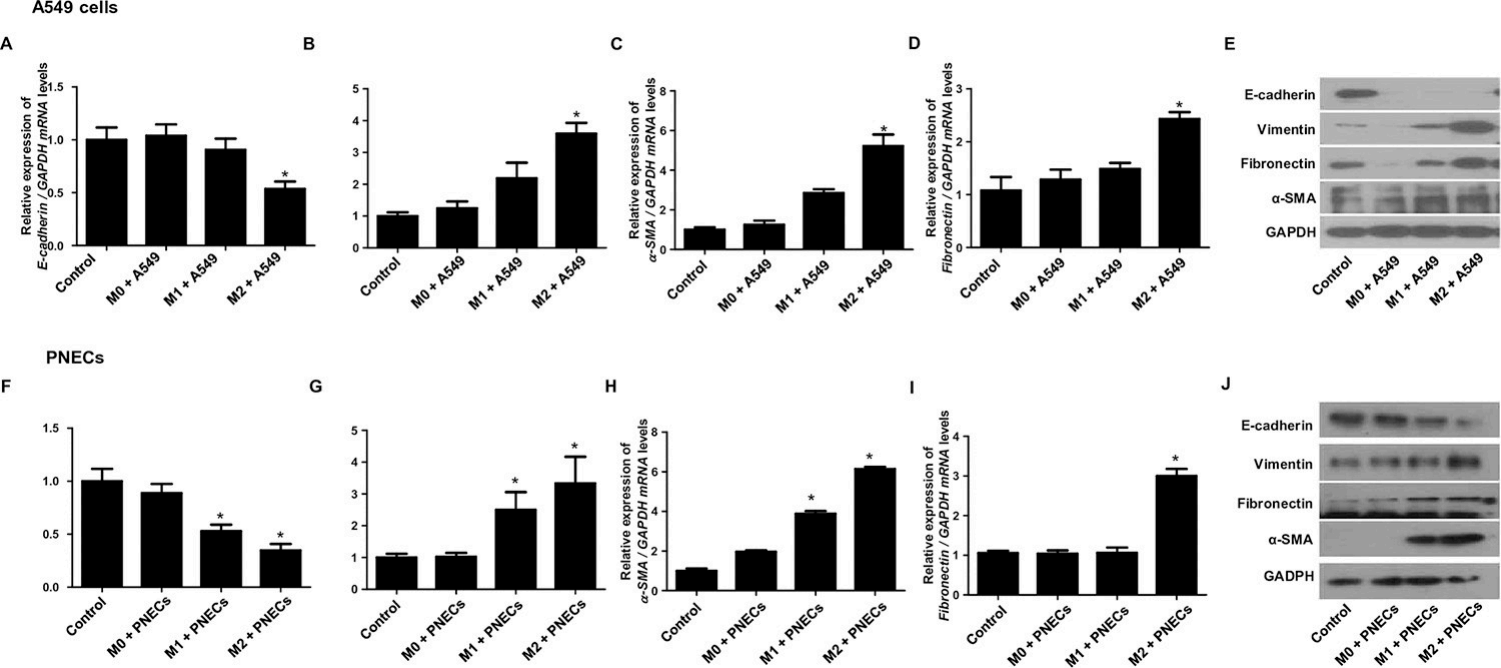
Polarized THP-1 macrophages induced EMT in epithelial. (A-D) Real-time PCR analysis of E-cadherin, Vimentin, α-SMA, and fibronectin mRNA expression in A549 cells cocultured with M0, M1, or M2 macrophages. (E) Western blot analysis of E-cadherin, Vimentin, α-SMA, and fibronectin protein expression in A549 cells cocultured with M0, M1, or M2 macrophages. (F-J) Real-time PCR and Western blot analysis of E-cadherin, Vimentin, α-SMA, and fibronectin mRNA and protein expression in PNEC cells cocultured with M0, M1, or M2 macrophages. Data are presented as mean ± SEM, *p<0.05 compared to control. Data are presented as the mean ± SD of three independent experiments.

To investigate the effect of polarized THP-1 macrophages on EMT of epithelial cell, coculture experiments were performed using THP-1 cells differentiated into M1, or M2 macrophages and A549 or PNECs. The results showed that coculture with M2 macrophages significant decreased E-cadherin mRNA expression and increased Vimentin, α-SMA, and fibronectin mRNA expression in A549 cells, compared to A549 cells cultured alone. No significant changes were observed in E-cadherin, Vimentin, α-SMA, or fibronectin mRNA expression in A549 cells cocultured with M0 or M1 macrophages (Fig 3A–3D). Similar results were observed at the protein level (Fig 3E). When cocultured with M2 macrophages, PNECs also showed a significant decrease in E-cadherin mRNA and protein expression, and an increase in Vimentin, α-SMA, and fibronectin mRNA and protein expression, similar to the results observed in A549 cells (Fig 3F–3J).

### rhMMP-12 induced EMT process in A549 cells and PNECs

**Fig 4:**
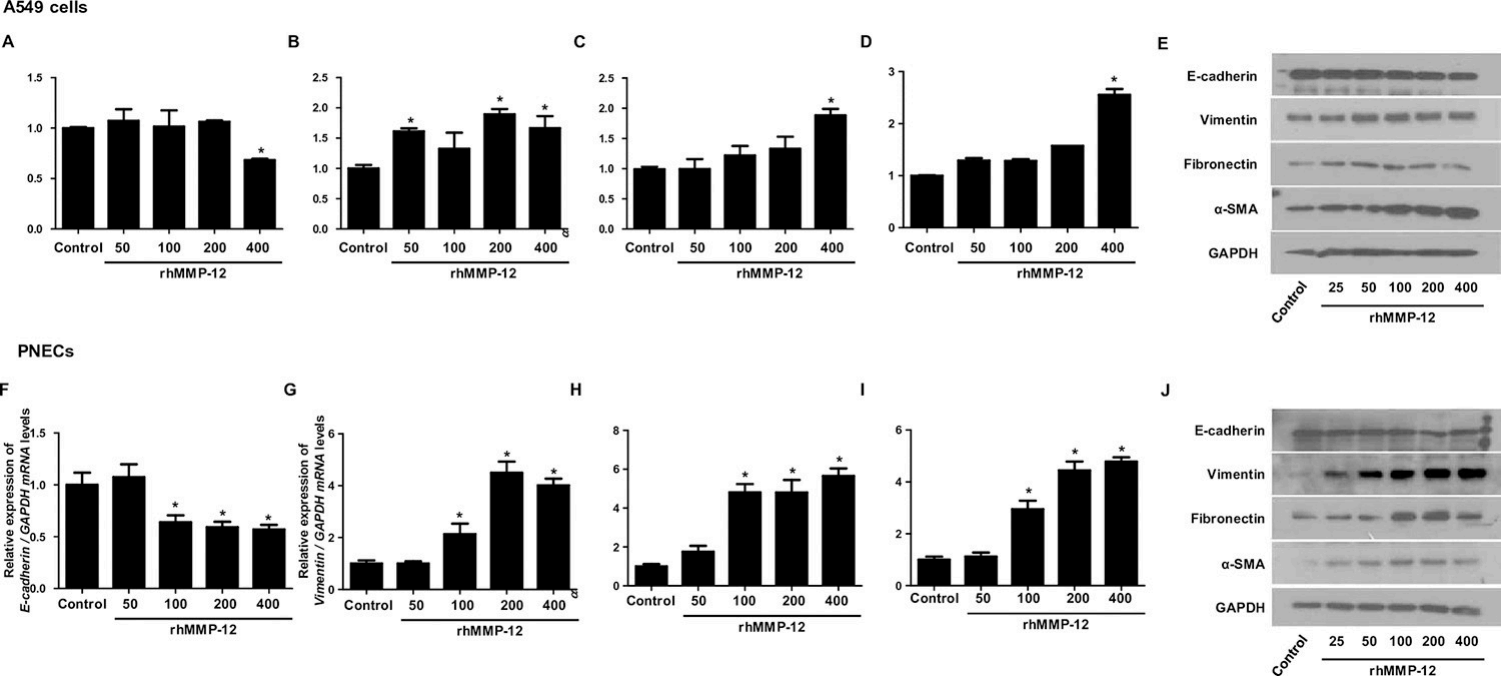
Treatment with recombinant MMP-12 induces EMT in A549 and PNEC cells. A549 cells treated with different concentrations of rhMMP-12 (0, 100, 200, and 400 nM). (A-D) Real-time PCR and (E) western blot analysis of E-cadherin, Vimentin, α-SMA, and fibronectin mRNA expression in A549 cells treated with rhMMP-12 at different concentrations. (F-J) Similar results were observed in PNEC cells treated with rhMMP-12. *p < 0.05 compared to the control. Data are presented as the mean ± SD of three independent experiments.

Recombinant MMP-12 (rhMMP-12), which is mainly produced by M2 macrophages, was treated at different concentrations to determine whether it induces EMT. The treatment of rhMMP-12 decreased E-cadherin and significantly increased Vimentin, α-SMA, and fibronectin mRNA and protein levels at a concentration of 400ng/ml in A549 cells (Fig 4A–4E). Similarly, the treatment of rhMMP-12 resulted in a significant decrease in E-cadherin levels and an increase in Vimentin, α-SMA, and fibronectin levels at a concentration of 100ng/ml in PNECs. The protein results in PNECs were consistent with these findings (Fig 4F–4J). These results suggest that MMP-12 plays a role in inducing EMT in both A549 cells and PNECs.

### siRNA MMP-12 inhibited M2 macrophage-induced EMT in PNEC cells and ALI culture

**Fig 5:**
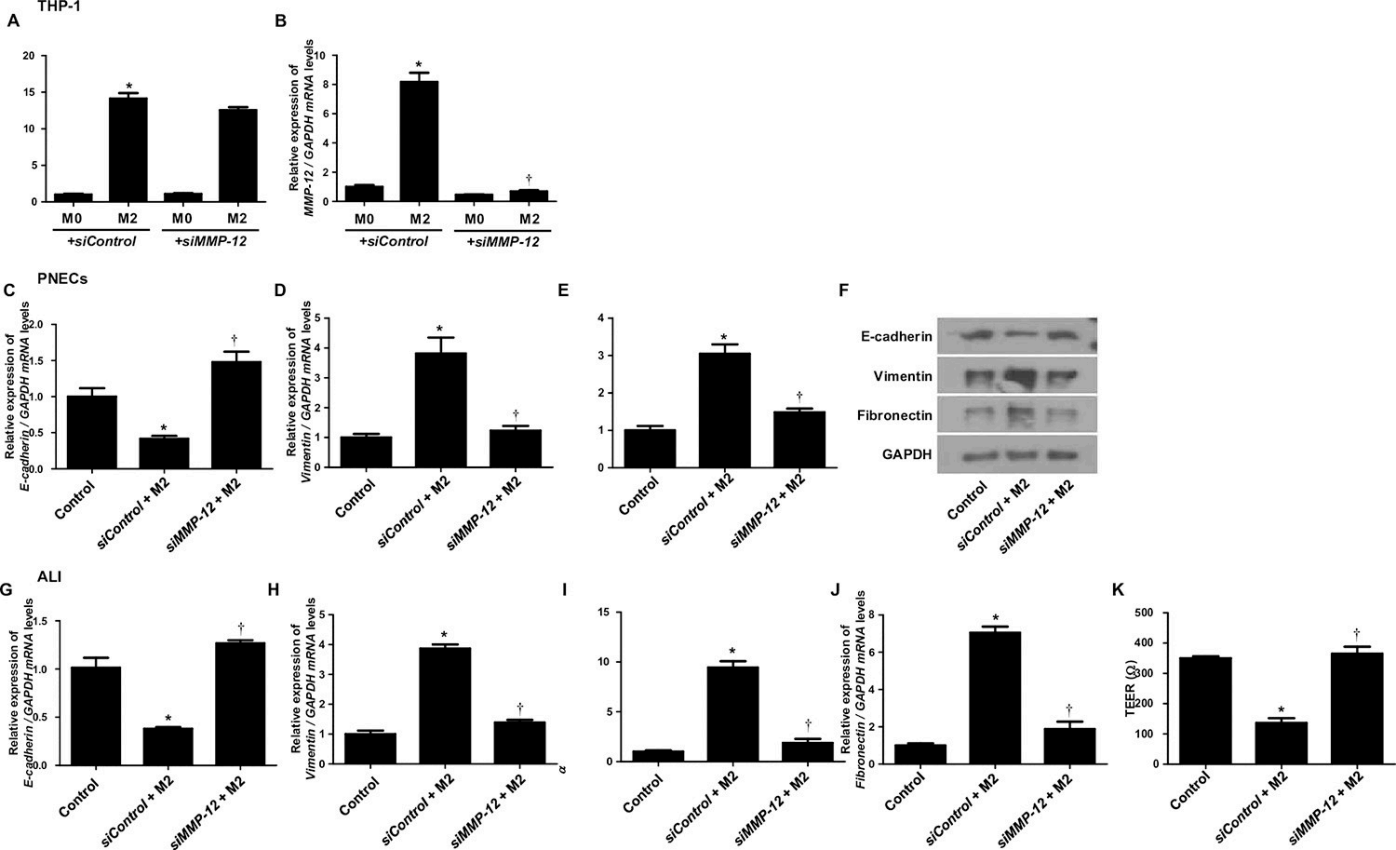
MMP-12 knockdown inhibits EMT induced by M2 macrophages. Real-time PCR analysis of (A) CD206 and (B) MMP-12 expression in M2 macrophages transfected with siMMP-12 or sicontrol. (C-E) Real-time PCR and (F) western blot analysis of E-cadherin, vimentin, and fibronectin mRNA and protein expression in PNECs cocultured with siMMP-12 or sicontrol-transfected M2 macrophages. (G-J) Real-time PCR analysis of E-cadherin, vimentin, and fibronectin mRNA expression in ALI culture cocultured with siMMP-12 or sicontrol-transfected M2 macrophages. (K) The effect of MMP-12 knockdown on the TEER of ALI culture. All data are presented as mean ± SEM. *p < 0.05 compared to the sicontrol group or control group. †p < 0.05 compared to the sicontrol group. Data are presented as the mean ± SD of three independent experiments.

To confirm whether MMP-12 is directly involved in EMT, siMMP-12 was used to knockdown MMP-12 in M2 macrophages and the effect on EMT was examined. The transfection of siMMP-12 did not change CD206 expression, but significantly inhibited MMP-12 expression, indicating successful knockdown (Fig 5A and 5B). This demonstrated that MMP-12 inhibition did not affect the differentiation of M2 macrophages. Coculturing PNECs with siMMP-12-transfected M2 macrophages and examining EMT showed that compared to the *sicontrol*, E-cadherin was increased, and vimentin and fibronectin were suppressed (Fig 5C–5F). To confirm these findings, siMMP-12 was also transfected into M2 macrophages and cocultured with ALI culture that mimics the biological characteristics of epithelial cells. The transfection of siMMP-12 increased M2 macrophage-inhibited E-cadherin and suppressed M2 macrophage-induced vimentin and fibronectin, similar to the results observed in PNECs (Fig 5G–5J). Additionally, the decrease in TEER caused by M2 macrophages was restored by siMMP-12 (Fig 5K). These findings suggest that MMP-12 plays a direct role in inducing EMT in nasal epithelial cells and siMMP-12 may be a potential therapeutic target for repairing epithelial barrier dysfunction.

### MMP408 inhibited MMP-12-induced EMT in PNEC cells and ALI culture

**Fig 6:**
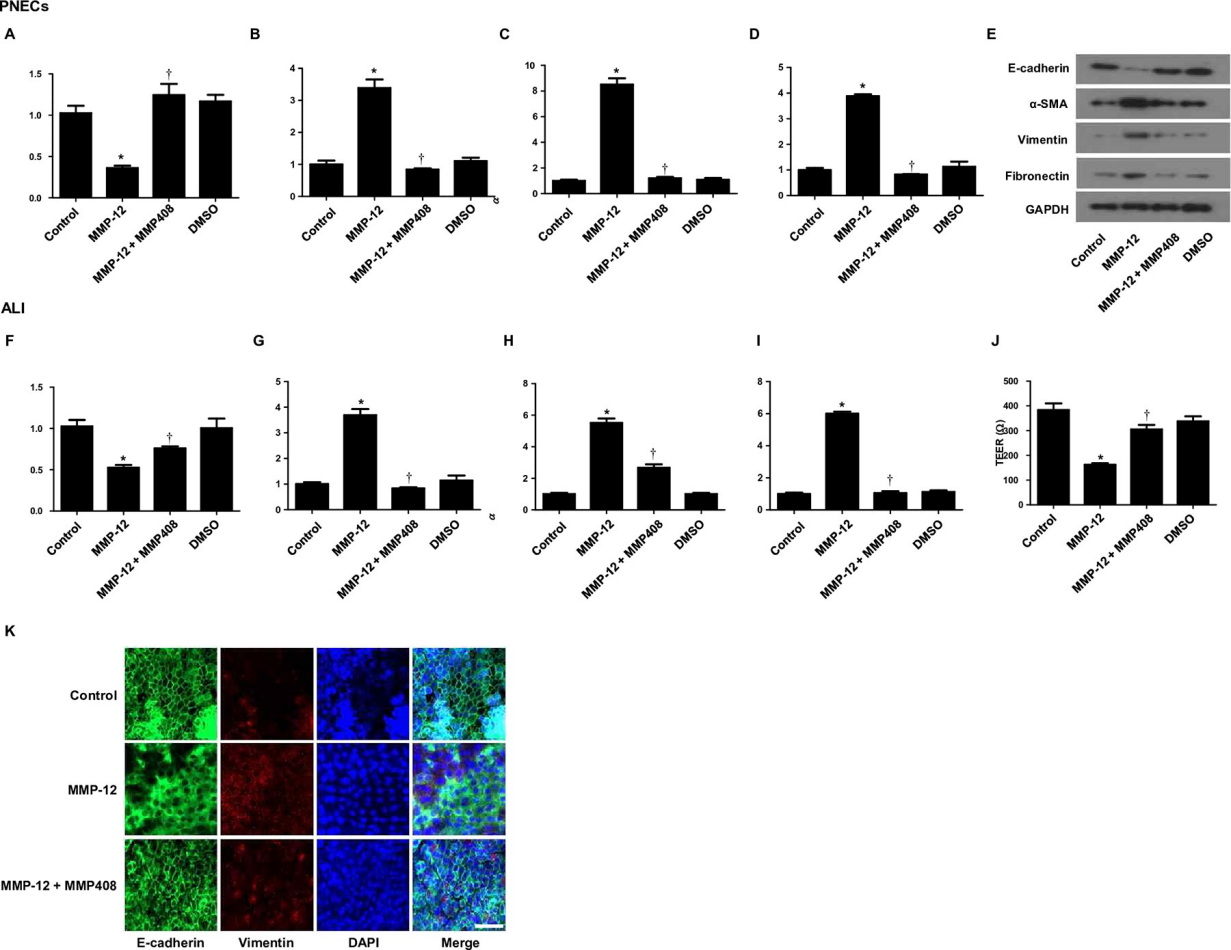
Inhibition of MMP-12 with MMP408 suppresses EMT in nasal epithelial cells. (A-C) Relative mRNA expression levels of E-cadherin, Vimentin, and α-SMA in PNECs treated with MMP408(10 nM) and/or rhMMP-12 (400 nM), as determined by Real-time PCR. (D-F) Relative protein expression levels of E-cadherin, Vimentin, and fibronectin in PNECs treated with MMP408 and/or rhMMP-12, as determined by Western blot analysis. (G-I) Relative mRNA expression levels of E-cadherin, Vimentin, and α-SMA in ALI cultures treated with MMP408 and/or rhMMP-12, as determined by Real-time PCR. (J) TEER values in ALI cultures treated with MMP408 and/or rhMMP-12. (K) Immunofluorescence analysis showing E-cadherin (green) and vimetin expression (red). *P< 0.05 compared to the control. †P< 0.05 compared to rhMMP12 treated group. Data are presented as the mean ± SD of three independent experiments.

We evaluated the inhibitory effect of MMP408, a known chemical inhibitor of MMP-12, on EMT. Treatment with MMP408 resulted in increased the mRNA and protein expression of E-cadherin, which was suppressed by MMP-12. Additionally, MMP408 treatment suppressed the mRNA and protein expression of Vimentin, α-SMA, and fibronectin, which were upregulated by MMP-12 (Fig 6A–6I). These findings were consistent in both PNECs and ALI cultures. Furthermore, treatment with MMP408 increased the decreased TEER value caused by MMP-12 in the ALI culture (Fig 6J).

## Discussion

The present study aimed to investigate the role of MMP-12 in inducing epithelial-mesenchymal transition (EMT) in chronic rhinosinusitis with nasal polyps (CRSwNP). Our results showed that MMP-12 was highly expressed in CD206-positive cells and CD163 positive cells in CRSwNP nasal tissue. rhMMP-12 induced EMT in both A549 cells and PNECs. MMP-12 knockdown by *siMMP-12* in M2 macrophages significantly inhibited EMT in PNECs and in an air-liquid interface (ALI) culture system. Moreover, treatment with an MMP-12 chemical inhibitor, MMP408, had similar effects on EMT inhibition in both PNECs and the ALI culture system.

From an immunological perspective, macrophages have been shown to contribute to the pathogenesis of various inflammatory diseases, including CRS [15]. Chronic rhinosinusitis (CRS) is a multifactorial disease with a complex immune response. Recent studies have suggested that macrophages play a critical role in the pathogenesis of CRS [7]. Macrophages are a heterogeneous population of cells that can be polarized into two main subtypes: M1 and M2. M1 macrophages are classically activated and produce pro-inflammatory cytokines, while M2 macrophages are alternatively activated and contribute to tissue repair and remodeling [16]. In our study, we focused on M2 macrophages since they have been shown to be more prevalent in CRS tissue. In particular, M2 macrophages are known to promote tissue repair and remodeling by producing growth factors and extracellular matrix components [17]. However, dysregulated M2 polarization has been linked to various pathological conditions, including cancer and fibrosis [11, 18]. Our study demonstrates that M2 macrophages in CRSwNP tissues express high levels of CD206, a marker of M2 polarization, and produce MMP-12, which contributes to EMT and tissue remodeling. Therefore, modulating macrophage polarization and function may represent a promising approach for treating CRSwNP. Future studies aimed at understanding the underlying mechanisms of macrophage activation and polarization in CRSwNP will further our knowledge of the disease and may lead to the development of novel therapeutic strategies.

Matrix metalloproteinase-12 (MMP-12) is a member of the matrix metalloproteinase family, which is involved in the degradation and remodeling of extracellular matrix (ECM) components [19]. MMP-12 is primarily expressed by macrophages and has been implicated in various physiological and pathological processes, including tissue remodeling, inflammation, and cancer [20, 21]. In the context of CRS, MMP-12 has been shown to play a role in tissue remodeling and EMT. EMT is a process by which epithelial cells lose their polarity and adhesion properties and acquire mesenchymal-like characteristics. This process is involved in tissue remodeling and repair but can also contribute to pathological processes such as fibrosis [12]. MMP-12 has been shown to promote EMT in airway epithelial cells by cleaving E-cadherin, a cell-cell adhesion protein, and inducing the expression of mesenchymal markers such as vimentin and fibronectin. Moreover, MMP-12 has been implicated in the pathogenesis of CRS by promoting tissue remodeling and angiogenesis. In a study of nasal polyps, a common complication of CRS, MMP-12 was found to be upregulated in the polyp tissues compared to healthy nasal mucosa [13]. Furthermore, MMP-12 was shown to promote tissue proliferation in vitro, suggesting that it may contribute to the development of nasal polyps in CRS.

Our findings strongly support the hypothesis that MMP-12 produced by M2 macrophages is a critical mediator of EMT in CRSwNP. By promoting the loss of epithelial characteristics and the acquisition of mesenchymal traits, MMP-12 facilitates tissue remodeling processes that are essential for polyp formation and disease persistence in CRSwNP. The evidence that MMP-12 expression is elevated in CRSwNP tissues and correlates with EMT markers suggests that it could serve as both a biomarker of disease severity and a potential therapeutic target. These findings align with previous studies that have demonstrated the role of MMP-12 in airway remodeling and fibrosis in other diseases, such as COPD and asthma [22]. Similar mechanisms may be involved in CRSwNP, where MMP-12 contributes to tissue remodeling and disease persistence. Targeting MMP-12 may help to reduce or prevent the pathological EMT and tissue remodeling observed in CRSwNP, thereby providing a novel approach to treating this chronic inflammatory condition [13, 21].

Translating these findings into clinical practice could involve developing selective MMP-12 inhibitors to reduce disease progression in CRSwNP patients. Moreover, given the significant role of MMP-12 in promoting EMT and tissue remodeling, targeting MMP-12 could be particularly beneficial in treating eosinophilic CRSwNP, where inflammation and tissue remodeling are more pronounced. Furthermore, MMP-12 may serve as a biomarker for disease severity, helping clinicians stratify patients and personalize treatment strategies. These approaches could significantly enhance the management of CRSwNP by providing targeted therapies that focus on the molecular mechanisms underlying disease pathology. Future clinical studies should evaluate the effectiveness and safety of MMP-12 inhibitors in treating CRSwNP.

One potential limitation of using macrophages differentiated from THP-1 cells is that they may not fully represent the phenotype and function of tissue-resident macrophages in vivo. THP-1 cells are a human monocytic cell line that can be differentiated into macrophages, but they lack the tissue-specific cues and signals that regulate macrophage differentiation and function in vivo [23]. Thus, the behavior of THP-1-derived macrophages may differ from that of macrophages derived from primary tissues, including the sinuses. Additionally, THP-1 cells may not accurately reflect the heterogeneity of macrophage populations present in vivo. However, THP-1 cells are used as an alternative to primary macrophages due to their abundant availability, ease of manipulation, and reproducibility in the laboratory setting. THP-1 cells offer a controlled and standardized model system, facilitating high-throughput studies and investigations into macrophage behavior and function [24].

In conclusion, our study demonstrates that M2 macrophages in CRSwNP tissues express high levels of CD206 and produce MMP-12, which contributes to tissue remodeling and EMT. These findings suggest that modulating macrophage polarization and function may represent a promising approach for treating CRSwNP. Future studies aimed at understanding the underlying mechanisms of macrophage activation and polarization in CRSwNP will advance our knowledge of the disease and may lead to the development of novel therapeutic strategies targeting MMP-12 and related pathways.

## Supporting information

S1 Raw data (XLSX)

S1 Raw images (PDF)
