# Supplementary figures and images for: Matrix metalloproteinase-12 by M2 macrophages induced epithelial to mesenchymal transition in chronic rhinosinusitis with nasal polyps
Source: PLoS One. 2024 Dec 31;19(12):e0313097. doi: 10.1371/journal.pone.0313097 (PMC11687655; doi:10.1371/journal.pone.0313097)

Figure 3

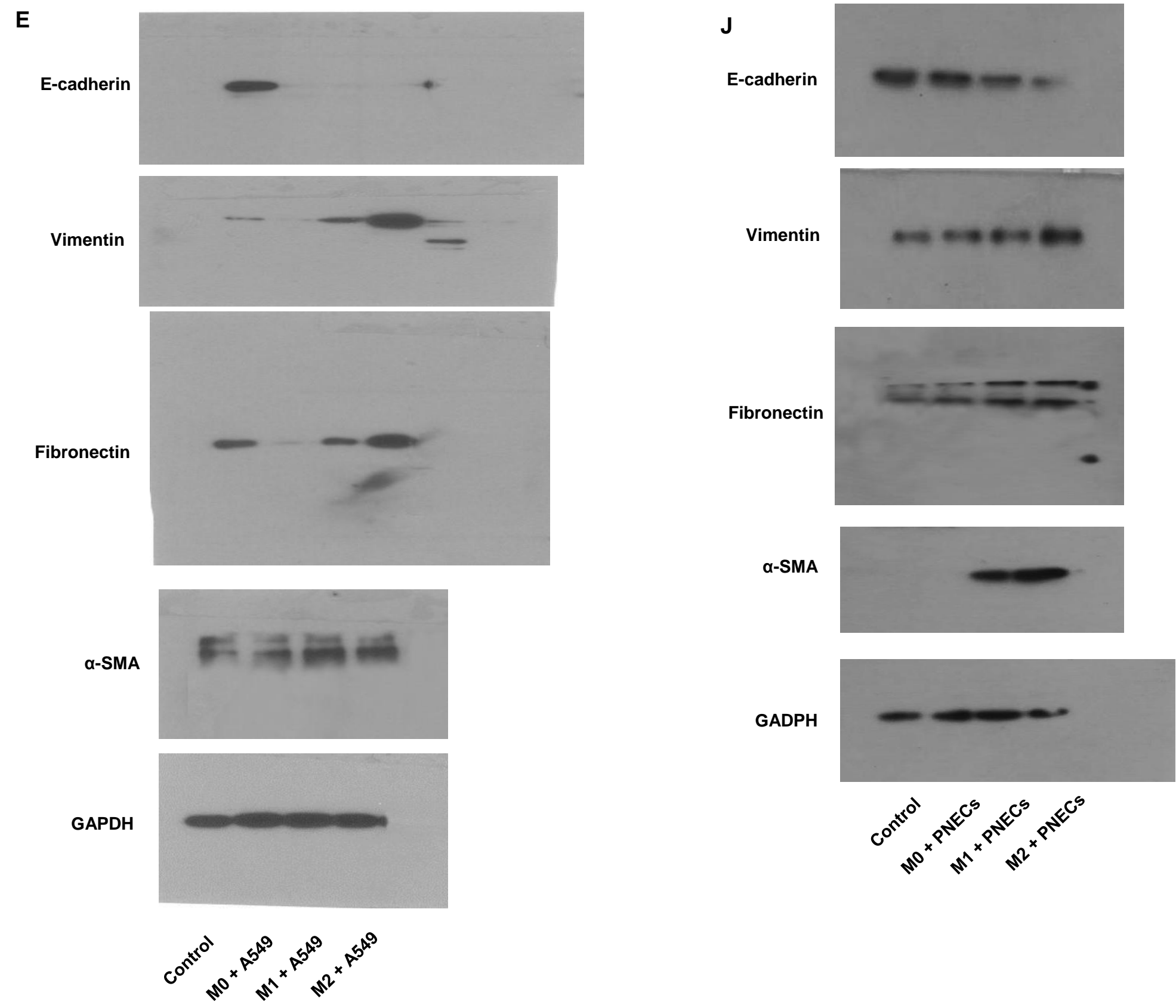

Figure 4

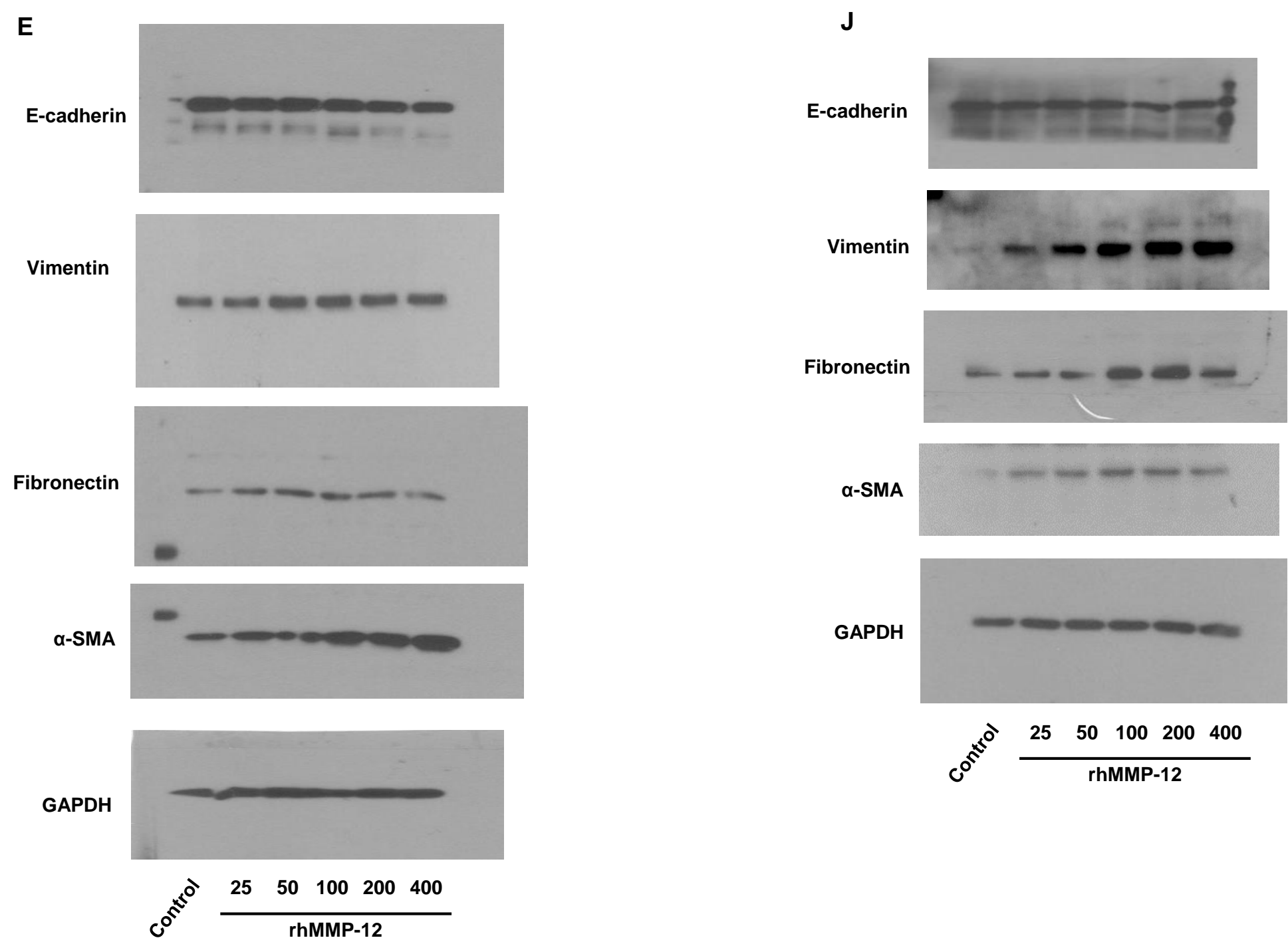

Figure 5

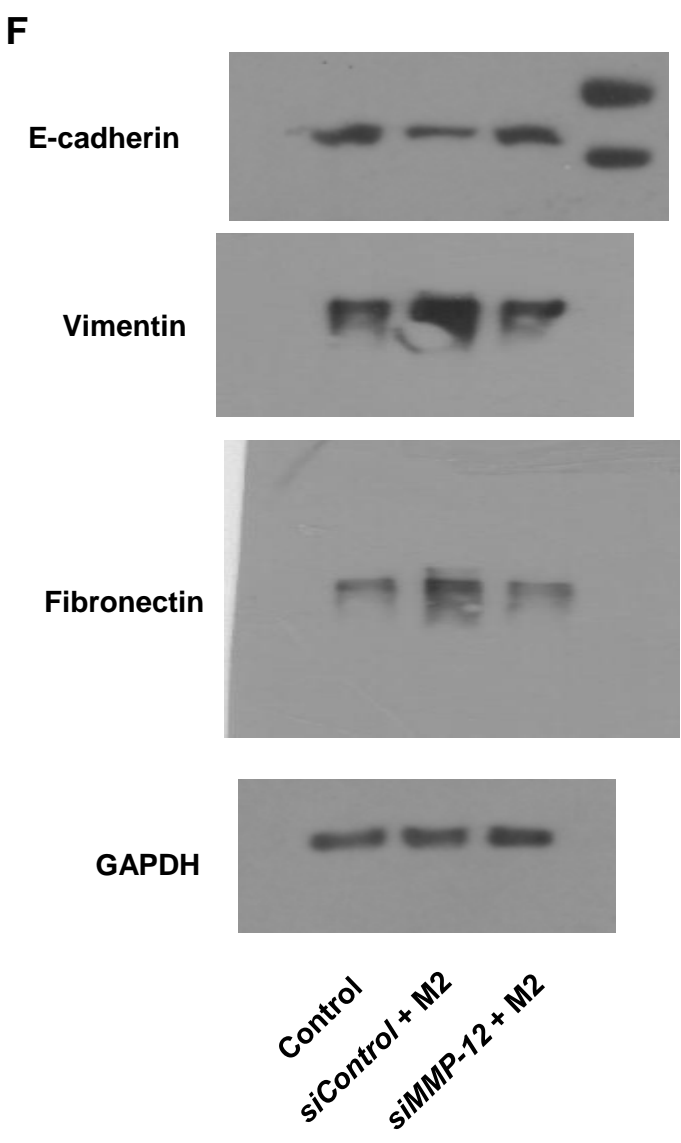

Figure 6

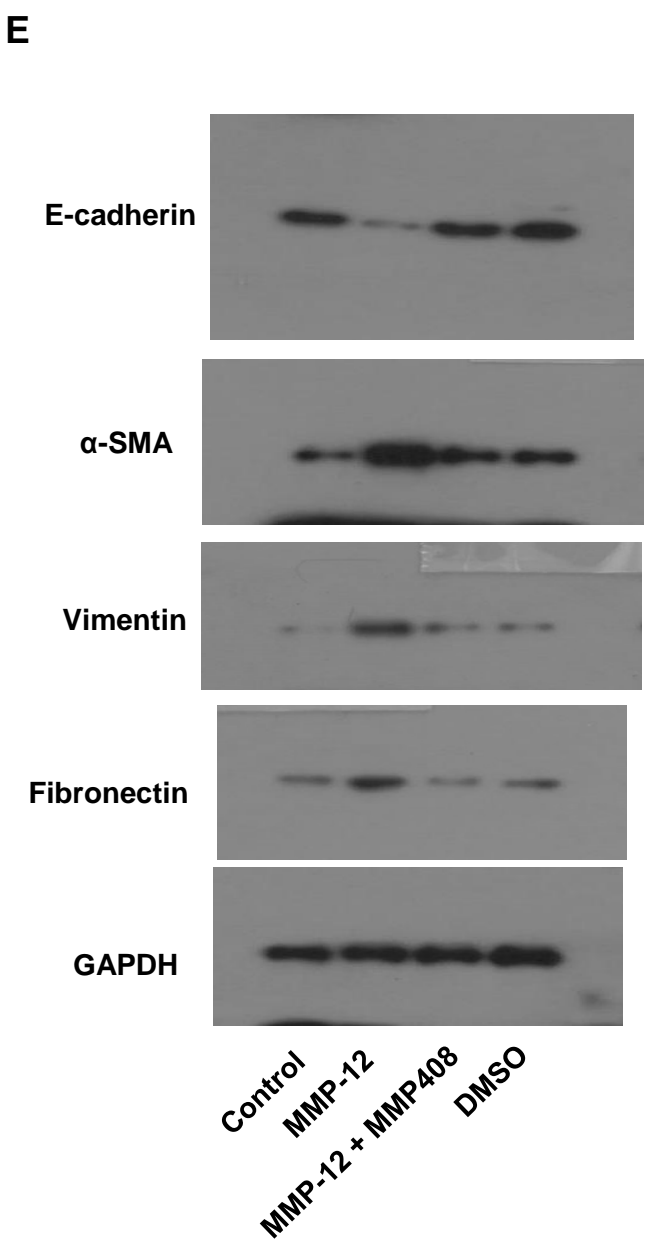

Supplement: S1 Raw images — (PDF) [file pone.0313097.s002.pdf]
